# Preoperative Thyrotropin Serum Concentrations Gradually Increase from Benign Thyroid Nodules to Papillary Thyroid Microcarcinomas Then to Papillary Thyroid Cancers of Larger Size

**DOI:** 10.1155/2012/530721

**Published:** 2011-07-19

**Authors:** Carles Zafon, Gabriel Obiols, Juan Antonio Baena, Josep Castellví, Belen Dalama, Jordi Mesa

**Affiliations:** ^1^Department of Endocrinology, Hospital General Universitari Vall d'Hebron, Pg. Vall d'Hebron 119-129, 08035 Barcelona, Spain; ^2^Department of Surgery, Unit of Endocrinological Surgery, Hospital General Universitari Vall d'Hebron, 08035 Barcelona, Spain; ^3^Department of Pathology, Hospital General Universitari Vall d'Hebron, 08035 Barcelona, Spain

## Abstract

We evaluated the preoperative serum thyrotropin (TSH) levels in 386 patients operated on for nodular thyroid disease (NTD). TSH levels for cases with final benign disease and differentiated thyroid carcinoma (DTC) were compared. No evidence of cancer was detected in 310 patients (80.3%), whereas malignancy was present in 76 cases (19.7%). Mean TSH concentration was 1.36 ± 1.62 mU/L in benign patients and 2.08 ± 2.1 in cases with malignant lesions (*P* = 0.0013). The group of malignancy was subdivided in papillary thyroid carcinoma (PTMC) versus thyroid cancer of larger size (TCLS). Mean TSH was 1.71 ± 1.52 in PTMC and 2.42 ± 2.5 in TCLS. Significant differences were found when all groups (benign, PTMC and TCLS) were compared (*P* < 0.001). However, pairwise comparisons between them showed that differences were only significant between benign and TCLS groups (*P* < 0.01). In conclusion, TSH levels were higher in patients with a final diagnosis of DTC. Moreover, it appears that there exists an increment in tumor size as a function of increment in the TSH level.

## 1. Introduction

Treatment of differentiated thyroid carcinomas (DTCs) includes total thyroidectomy, followed by removing affected (and nonaffected) lymph nodes in the central compartment of the neck, radioiodine for ablation of thyroid remnants or metastases, and suppressive treatment with L-thyroxine [1]. A supraphysiologic dose of thyroid hormone to suppress the secretion of endogenous thyroid-stimulating hormone (TSH) is associated with a longer relapse-free survival, overall survival, and carcinoma-related death [2]. The rationale for this approach is that TSH is the main regulator of thyrocyte differentiation and growth, and this capacity is retained in cancer cells of DTCs [3].

Furthermore, in recent years it has been shown that serum TSH concentration is an independent predictor of thyroid malignancy [4]. Several clinical studies have reported that higher levels of TSH are associated with an increased incidence of thyroid cancer in patients with nodular thyroid disease (NTD). It is currently not clear whether TSH is involved in the development of thyroid cancer, in the progression of thyroid cancer, or both [5]. Two reports have demonstrated that higher preoperative serum TSH levels are associated with more advanced cancer stages at the time of diagnosis [6, 7]. Moreover, Gerschpacher et al. [8] found that the TSH level is not elevated in subjects with papillary thyroid carcinoma (PTC) measuring ≤1 cm in size, a subset of tumors referred to as papillary thyroid microcarcinomas (PTMCs). Together, these findings support the notion that TSH may contribute to tumor progression. 

The present study was conducted to determine the preoperative serum TSH levels in a group of patients who underwent surgery for NTD. TSH concentrations were correlated with the final histological diagnosis, defined as the presence or absence of malignancy. Moreover, the group of malignancies was subdivided between patients with PTMCs and patients with thyroid cancers of larger size (TCLS).

## 2. Material and Methods

From January 2006 to December 2009, 438 patients underwent thyroid surgery for NTD. Patients (i) with known thyroid cancer, (ii) without an available serum TSH concentration within 1 year before surgery, (iii) with a final histological diagnosis other than DTC (e.g., medullary thyroid cancer or anaplastic thyroid cancer), and (iv) hyperthyroidism due to the Graves disease were not included. Three hundred eighty-six patients were eligible for the study.

All patients had a solitary thyroid nodule or a multinodular goiter detected by clinical examination, ultrasound scan (US), or both. Preoperatively, thyroid USs confirmed NTD in all cases. A fineneedle aspiration biopsy (FNAB) was performed for thyroid nodules > 1 cm, or nodules < 1 cm with suspicious US features. Thyroidectomy was prescribed for patients with malignant, suspicious, or repetitive indeterminate nodules according to FNAB results. Moreover, surgery was indicated for benign disease when local symptoms were present or for esthetic reasons. 

All patients had a serum TSH level within 1 year before surgery measured by an automated immunochemiluminescent assay (Immulite 2500; Siemens, Los Angeles, Calif, USA). The normal range was 0.4–4.0 mU/L. 

Statistical analysis was performed to determinate whether or not there were differences in age and TSH levels between patients diagnosed with benign lesions, compared with those diagnosed with thyroid cancer. Furthermore, the group of patients with malignancies was subdivided into PTMCs and TCLS. Age was evaluated as a continuous variable. The TSH concentration was evaluated as a continuous variable and categorically within the following 3 ranges: <0.4 mU/L (subclinical hyperthyroidism); 0.4–4.0 mU/L (euthyroidism); >4.0 mU/L (subclinical hypothyroidism). The Student's *t*-test was used for numerical variables. The Fisher's exact test was used for categorical variables. The nonparametric Kruskall-Wallis test was used to determinate whether there were significant differences between patients with benign, PTMCs, and TCLS. Afterwards, Bonferroni's adjustment for multiple comparisons was applied to the pairwise comparisons of groups. Values were reported as the mean ± SEM. A *P* value < 0.05 was considered statistically significant.

## 3. Results

There were 386 patients who met the inclusion criteria. The final pathology data showed no evidence of malignancy in 310 patients (80.3%), whereas malignant lesions were present in 76 cases (19.7%). 

The benign group included 250 females (80.6%) and 60 males (19.4%). The mean age at the time of diagnosis was 54.4 ± 14.2 years. There were 247 (79.7%) patients with multinodular goiter and 63 (20.3%) patients with thyroid nodule. Histological features of chronic thyroiditis were present in 75 (24.2%) patients. 

 DTC was diagnosed in 60 females (78.9%) and 16 males (21.1%). The mean age at the time of diagnosis was 49.3 ± 14.4 years. There was a significant difference in age between both groups (*P* = 0.005). There were 45 (59.2%) patients with multinodular goiter and 31 (28.2%) patients with thyroid nodule. Histological features of chronic thyroiditis were present in 18 (23.7%) patients. In the cohort of patients with malignancies, PTMCs were demonstrated in 36 patients (47.3%) and TCLS in the remaining 40 patients (PTC, *n* = 37; follicular thyroid carcinoma, *n* = 3; mean tumor size 25.21 ± 11.8 mm). The mean age in the patients with PTMCs was 52.1 ± 14.7 years, and 46.8 ± 13.8 years in the patients with TCLS.

**Figure 1:**
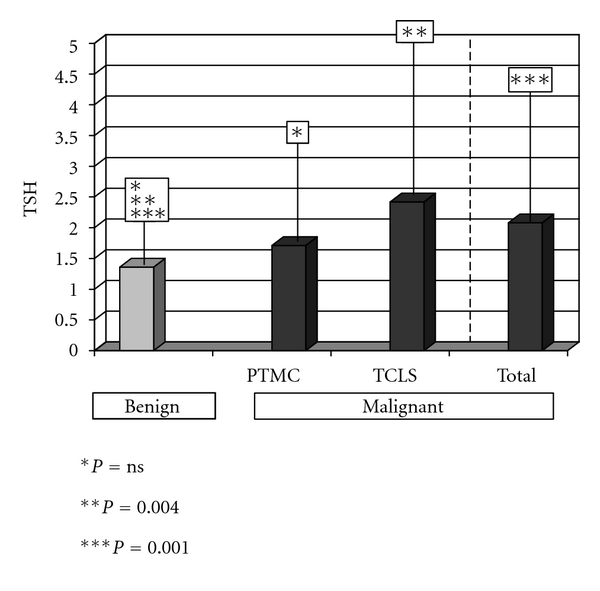
TSH concentrations in the different study groups. PTMC: papillary thyroid microcarcinoma and TCLS: thyroid carcinoma of larger size).

Figure 1 shows the TSH concentrations in the different study groups. The preoperative mean TSH level in the 310 patients with no evidence of malignancy was 1.36 ± 1.62 mU/L, whereas the mean TSH concentration in the group of 76 patients with malignancies was 2.08 + 2.1 mU/L (*P* = 0.0013). Thus, the TSH levels were higher in patients with a final diagnosis of DTC. Among the set of patients with malignant nodules, mean TSH was 1.71 ± 1.52 mU/L in patients with PTMCs and 2.42 ± 2.5 mU/L in patients with TCLS. Statistical significance was found when the three groups (benign, PTMCs, and TCLS) were compared (*P* < 0.001). However, pairwise comparisons between them showed that differences in TSH were only significant between benign and TCLS groups (*P* < 0.01).

Seventy-four (23.9%) patients with benign nodules were classified as subclinical hyperthyroidism (TSH < 0.4 mU/L). This diagnosis was only detected in 10 patients (13.1%) with malignancies (*P* = 0.027). In contrast, 8 patients (10.6%) with thyroid cancer were diagnosed with subclinical hypothyroidism (TSH > 4 mU/L), whereas this profile existed in 11 patients (3.5%) with benign lesions (*P* = 0.01). In summary, in patients with subclinical hyperthyroidism, normal TSH concentration and subclinical hypothyroidism malignancy rate were 12%, 20.5%, and 42%, respectively, (Table 1). 

When we excluded all patients with TSH out of the normal range, the association between the TSH level and the final histology remained significant. The mean TSH level in the 225 patients with no evidence of malignancy and a normal TSH was 1.47 ± 0.8 mU/L, whereas the mean TSH level in the 58 patients with malignancies was 1.8 ± 0.8 mU/L (*P* = 0.009). Among this last group, mean TSH level was 1.75 ± 0.8 mU/L in patients with PTMCs and 1.84 ± 0.9 mU/L in patients with TCLS. Again, these differences were not significant. However, the TSH levels were significantly different between the group with benign lesions and a normal TSH level and patients with TCLS and a normal TSH level (*P* = 0.026). Finally, there were not significant differences among groups related to the presence of chronic thyroiditis.

## 4. Discussion

Several epidemiologic studies have confirmed that during the last decade, the incidence of DTC, and especially PTC, has increased worldwide [9, 10]. Furthermore, this highest incidence has been observed in tumors <1 cm in size, that is, PTMCs [11]. PTMCs exhibit significant differences in the mode of presentation from papillary tumors of larger size. Although PTMCs exhibit a more benign behavior [12], some authors suggest that there exists a subgroup of PTMCs that can be aggressive, requiring therapeutic management similar to larger tumors. 

Boelaert et al. [4] reported, for the first time, that the TSH serum concentration could be an independent predictor of thyroid malignancy. The authors found that the risk of diagnosis of DTC rises in parallel with TSH serum levels. Moreover, they derived a formula for risk of malignancy based on the TSH level. Thereafter, these findings were subsequently confirmed by others. Haymart et al. [7] found that the preoperative mean TSH level is significantly higher in patients with a final diagnosis of thyroid cancer. The same group has shown that this relationship is independent from age [13]. Jonklaas et al. [14] reported that this association remained in a strictly euthyroid population (after subclinical hypo- and hyperthyroidism cases were excluded) who underwent thyroid surgery for NTD. In this regard, Polyzos et al. [15] confirmed that TSH levels are predictive of malignancy only within a normal range because they did not find this association in patients with subclinical hypothyroidism. Jin et al. [16] have found that in patients with NTD, a serum TSH level < 0.9 has a probability of malignancy of approximately 10%, whereas in those patients with a serum TSH > 5.5, the rate of cancer is 65%. Recently, in a large series, Fiore et al. [17] reported that patients with nodular goiter treated with L-thyroxine had a significantly lower prevalence of PTC diagnosed by cytology. Moreover, the same authors reported that TSH levels were higher in patients with PTC than in benign NTD [6].

Two reports have demonstrated that a higher preoperative serum TSH was not only associated with the risk of DTC, but also associated with a more advanced stage of cancer at the time of diagnosis [6, 7]. In contrast, the association between TSH levels and PTMCs has not been extensively analyzed. In the series by Haymart et al. [7], the authors performed an analysis of the subset of tumors < 1 cm in size. Although the number of cases was very low, an increased risk of cancer persisted with a higher TSH in patients with PTMCs until the TSH was ≥ 5.00 mU/L. Recently, Gerschpacher et al. [8] compared the preoperative serum TSH concentrations between a cohort of 33 patients with PTMCs and a control group of 54 patients in which the thyroid gland was removed for medullary thyroid carcinoma or C-cell hyperplasia. The authors found no significant differences and concluded that the TSH level is not elevated in patients with PTMCs.

To our knowledge, the present paper is the first study that has not only compared TSH concentrations in patients with benign and malignant lesions, but also in PTMCs and TCLS. We have confirmed that the TSH level is significantly different between benign nodules and DTCs. These differences have persisted when comparing benign lesions and carcinomas > 1 cm in size, and when comparing all three groups. It appears that there exists an increment in tumor size as a function of increment in the TSH level. Thus, benign lesions have the lowest TSH levels, PTMCs have intermediate concentrations, and DTCs of larger size are associated with the highest levels of TSH. However, though the escalatory increment of TSH between the three groups of patients is evident, statistical analysis shows that differences are not significant, probably due to the small number of cases with cancer. According to these results, we suggest that TSH participates in the carcinogenesis of PTC, it is possibly that it acts as a growth factor and could be one of the parameters that determines the size of DTCs. In this regard, recently, Franco et al. [18] have found that TSH signaling pathway may predispose thyroid cells to BRAF-induced transformation, in mice with a thyroid-specific knockin of oncogenic *Braf* (LSL-*Braf* ^V600E^/TPO-*Cre*). 

 It is interesting to note that in our cohort, all patients underwent thyroid surgery. Thus, diagnoses of DTCs or benign thyroid disease were based on surgical pathologic results in all of the cases analyzed. In contrast, the diagnosis was established with FNAB results in other series without confirmatory histological results. For example, in the study by Boelaert et al. [4], only 37% of patients had definitive pathologic results. The final histological results were available in 21.6% of the patients in the series of Polyzos et al. [15]. Thus, in those series, it is possible that all of the microcarcinomas were not detected.

Unfortunately, information about thyroid antibodies was not analysed because there were no data in approximately one quarter of patients. However, histological preparations were examined, in order to verify the presence or not of autoimmune chronic thyroiditis. In this regard, we did not observe any differences between groups. 

Finally, it is well known the predominantly benign nature of thyroid autonomous nodules. Moreover, in patients with autonomous nodules, TSH concentrations were decreased or suppressed. In our study, the association between TSH level and final histology remained significant after patients with subclinical hypo- and hyperthyroidism were excluded. 

In summary, we have shown that TSH levels were higher in patients with a final diagnosis of DTC. Moreover, it appears that there exists an increment in tumor size as a function of increment in the TSH level. Although the role of the TSH level in the development and/or progression of thyroid cancer remains controversial, our results support the hypothesis that the TSH level might be involved in the progression, that is, the size, of an existing DTC.

## 5. Conflict of Interests

Authors declare no conflict of interests.

## Figures and Tables

**Table 1:** Distribution of patients according to TSH categories: <0.4 mU/L (subclinical hyperthyroidism); 0.4–4.0 mU/L (euthyroidism); >4.0 mU/L (subclinical hypothyroidism). PTMC: papillary thyroid microcarcinoma and TCLS: thyroid carcinoma of larger size.

TSH levels (mU/L)	Benign	PTMC	TCLS	Total malignant	All cases
<0.4	74	6	4	10	84
0.4–4.0	225	28	30	58	283
>4.0	11	2	6	8	19
